# Anxiety and depression mediate the relationship between digestive tract conditions and oral health-related quality of life in orthodontic patients

**DOI:** 10.3389/fpsyg.2022.873983

**Published:** 2022-07-29

**Authors:** Xue Tian, Yuan-hong Li, Lan-zhi Deng, Wen-ze Han, Dan Pu, Xiang-long Han, Shu-fang Du, Wei Deng

**Affiliations:** ^1^State Key Laboratory of Oral Diseases, National Clinical Research Center for Oral Diseases, West China Hospital of Stomatology, Sichuan University, Chengdu, China; ^2^School of Stomatology, Shanxi Medical University, Taiyuan, China; ^3^Department of Orthodontics, West China Hospital of Stomatology, Sichuan University, Chengdu, China; ^4^Affiliated Mental Health Center, Zhejiang University School of Medicine, Hangzhou, China; ^5^Hangzhou Seventh People’s Hospital, Zhejiang University School of Medicine, Hangzhou, China

**Keywords:** depression, anxiety, oral health, digestive tract condition, mediation analysis

## Abstract

**Background:**

Anxiety and depression are common psychological problems in orthodontic patients whose diet habits and oral health status change frequently during treatment. However, relationships between anxiety and depression, digestive tract condition, and impaired oral health-related quality of life remain unknown.

**Materials and methods:**

In this study, clinical assessments, including anxiety, depression, digestive tract condition, and oral health-related quality of life, were collected from 769 outpatients in the orthodontic department using three self-reported questionnaires. Correlation analysis was used to investigate the relationships among different clinical assessments. A chained mediation analysis model was further conducted to explore the direct and indirect effects of these various clinical factors.

**Results:**

Changes in digestive tract conditions were positively correlated with the psychological status and oral health-related quality of life. Anxiety and depression partially mediated the relationship between them, and the indirect effect was 0.68 (30%), of which the mediation effect of anxiety accounted for 56%.

**Conclusion:**

Anxiety and depression mediate the relationship between gastrointestinal conditions and oral health. In particular, anxiety seems to play a significant mediating role. Our findings indicate that psychological status must be paid more attention to in future clinical practices and supervision for digestive tract symptoms of orthodontic patients.

## Introduction

Anxiety and depression are common psychological disorders often observed during orthodontic treatment. The psychological health of orthodontic patients is of great concern because of its enormous effect on the oral health-related quality of life (OHRQoL), therapeutic effect, and satisfaction (Zhou et al., 2014; Choi et al., 2017; Aksoy et al., 2019).

Oral health-related quality of life is an essential index to measure the treatment effects and quality of life of orthodontic patients. The orthodontic treatment is used to correct malocclusion through orthodontic appliances, whose process is characterized by long-term, substantial changes in dietary habits and difficulty in maintaining oral hygiene. It makes patients more prone to digestive symptoms and emotional problems than ordinary people, decreasing their quality of life (Ren et al., 2014). Studies have reported that the application of orthodontic appliances led to a reduction in OHRQoL and mental health conditions during the first month, and the OHRQoL and psychological status recovered after orthodontic treatment (Johal et al., 2015; Abreu et al., 2018). With a lack of sufficient food intake, patients with malocclusion have higher gastrointestinal symptoms and lower gastric emptying rates (Suzuki et al., 2018). Additionally, the masticatory function declines due to the pain induced by orthodontic appliances (Magalhães et al., 2014; Shim et al., 2019; Miura et al., 2021), influencing the food intake of orthodontic patients with more fat and less fiber intake (Shirazi et al., 2011). Togawa et al. (2009) revealed that patients with malocclusion exhibited a higher incidence of gastrointestinal disorders, including gastroesophageal reflux disease.

Anxiety and depression are prevalent, but the relationship between gastrointestinal symptoms, psychological problems, and oral health is unknown. Gastrointestinal disorders interact with psychological and psychosomatic disorders, such as stress, depression, anxiety, and sleep disturbance (Konturek et al., 2011; Gracie et al., 2019; Lei et al., 2019). A meta-analysis demonstrated that patients with gastrointestinal diseases, such as irritable bowel syndrome, have remarkably higher levels of anxiety and depression than normal samples (Zhang et al., 2018), and gut microbiota is related to abnormal psychological states (Aleksandrova et al., 2017; Foster et al., 2017; Heym et al., 2019; Lukić et al., 2019; Van Ameringen et al., 2019). In addition, Valles-Colomer et al. (2019) identified covariations among the microbiome, quality of life, and depression and demonstrated that the microbiome significantly influences the quality of life and depression. All the evidence revealed a close relationship between psychological health status, orthodontic outcomes, and the OHRQoL in orthodontic patients.

Based on the above findings, we observed that mental health issues, such as anxiety and depression, play an essential role in somatic digestive symptoms and OHRQoL in orthodontic patients. However, the majority of studies did not elucidate the relationship among these factors, especially the indirect effect of psychological factors on the other two. In addition, many of them use small samples (Yuanyuan et al., 2014; Hongliang et al., 2015). According to the studies by Togawa et al. (2009); Ren et al. (2014), Valles-Colomer et al. (2019); Van Ameringen et al. (2019), we hypothesized that anxiety and depression mediate the relationship between gastrointestinal status and OHRQoL in orthodontic patients. In our study, with a large sample, we aimed to explore whether orthodontic treatment affects psychological status, digestive tract condition, and OHRQoL in orthodontic patients. Moreover, a mediation analysis was performed to elucidate the relationship between psychological status, digestive tract condition, and OHRQoL.

## Materials and methods

### Subjects

This study was approved by the Human Research Ethics Committee of Sichuan University (WCHIRB-D_2020-209). Before participation, the patients received a detailed explanation of the study approved by West China Hospital of Stomatology of Sichuan University. The patients’s verbal informed consents were recorded in questionnaires by filling in their names.

A cross-sectional survey was conducted among the Department of Orthodontics of West China Hospital of Stomatology outpatients at Sichuan University. A total of 769 patients (256 male and 513 female) meeting the following criteria were included to fill in self-reporting questionnaires: (1) patients diagnosed as Class I/II/III malocclusion in the first time visit and (2) patients diagnosed as Class I/II/III malocclusion undergoing orthodontic treatment. Patients treated with removable appliances are excluded. The following are two reasons why orthodontic patients with the primary gastrointestinal disease have not been excluded: First, it is difficult for patients themselves to identify whether they are primary or not through a self-reported investigation. Second, a cross-sectional study with large samples can partly reduce bias.

### Assessment instruments

#### Evaluation of gastrointestinal symptoms

The evaluation of gastrointestinal conditions was conducted by extracting the data of digestive tract-related items on the Patient Health Questionnaire (PHQ-15-D), including stomach pain, constipation, loose bowels, diarrhea and nausea, and gas or indigestion. We used PHQ-15 to evaluate gastrointestinal conditions because it has been widely used in clinical practice to measure somatic symptoms, and its self-report interval is in coordination with orthodontic treatment recheck duration. The validity and reliability of the Chinese version of PHQ-15 were evaluated at West China Hospital of Sichuan University (Cronbach’s alpha = 0.83) (Zhang et al., 2016). Patient’s scores for the items on a 3-point scale were based on the severity of the symptoms during the past 4 weeks (“0” = “not bothered at all,” “1” = “bothered a little,” and “2” = “bothered a lot”). No gastrointestinal symptoms group was defined as all items were scored as zero; otherwise, they were grouped into the gastrointestinal symptoms group.

#### Oral health impact profile-14

The short form of the oral health impact profile (OHIP-14) was coined and verified by measuring seven dimensions of oral health-related well-being, including functional limitation, physical pain, psychological discomfort, physical disability, psychological disability, social disability, and physical disabilities (Slade, 1997). The Chinese version of OHIP-14 was developed by Weini and Junqi (2006), exhibiting excellent reliability and validity (Cronbach’s α = 0.93). Each item of OHIP-14 is scored from 0 to 4, reflecting the occurrence from “never” to “very often.” The score of OHIP-14 reflects the level of OHRQoL. The lower total score is recognized as a better OHRQoL.

#### Hua-Xi emotional-distress index

Several scales are present to measure mental health. We chose HEI over PHQ9 or other scales because HEI is a local scale made by the Mental Health Center of West China Hospital that can evaluate anxiety and depression levels and has been used for years. HEI was developed in 2014 by a multidisciplinary team from West China Hospital of Sichuan University for screening depression and anxiety in non-psychiatric clinical settings, and the reliability and validity of HEI have been verified in West China Hospital [Cronbach’s α = 0.90, area under ROC curve (AUC) = 0.88 comparing with MINI interview] (Wang et al., 2017). The self-report questionnaire consists of nine items about emotional distress, including core symptoms of depression and anxiety; five items are associated with depression, and the remaining four items are associated with anxiety. The score of each item is ranked from 0 to 4 according to the occurrence frequency of the emotional experience in the recent month (“0” equals “Never”; “1” equals “Occasionally”; “2” equals “Some of the time”; “3” equals to “Most of the time”; “4” equals to “Nearly all the time”; respectively). The outcome is ranked using the total score: ≤8 (No negative emotions), 9–12 (Mild negative emotions), 13–16 (Moderate negative emotions), and ≥17 (Severe negative emotions).

### Statistical analysis

Statistical values were represented as “mean ± SD” and analyzed using conventional statistical analysis software (SPSS version 23 for Windows). *P*-value < 0.05 was considered statistically significant (*P < 0.05, ^**^P < 0.01, ^***^P < 0.001).

Descriptive statistics were conducted to summarize sample characteristics (age, sex, treatment duration, alcohol drinking, and appliance types). According to HEI and PHQ15-D scores, data were divided into subgroups in scale analysis. Student’s *t*-tests were used to compare means between subgroups. Correlation analysis was investigated with Spearman’s correlation coefficient to study the relationship among three scales.

#### Mediation analyses

To investigate the direct and indirect effects of anxiety and depression on scales with correlations, a chained mediation model was used. We used a plug-in called Process in SPSS software (version 23 for Windows) to assist in the construction of the chain mediator model, based on the relevant theoretical assumptions. In the chained mediator model, we denoted anxiety as mediator variable M1, depression as mediator variable M2, PHQ15-D as an independent variable X, and OHIP-14 as a dependent variable Y. The model controls for age and gender as covariates. Regression analysis was performed for every two variables to obtain different effect coefficients (e1, e2, e3, …), and the indirect effect coefficient values were further calculated for the different pathways. The total indirect effect value is the sum of the indirect effect values of all pathways. The total effect value is the sum of the total indirect effect value and the direct effect value. The direct or indirect effect was statistically significant when *P* < 0.05, and the 95% confidence interval did not include 0.

## Results

### Demographic information

The mean age of the current sample (*N* = 769) was 23.09 years (SD = 9.55). Most participants were female (*N* = 513, 66.7%). According to the HEI scale, 13.2% patients had mild emotional problems (*N* = 101), 2.2% had moderate emotional problems (*N* = 17), and 2.6% had severe emotional problems (*N* = 20). According to the PHQ15-D scale, 43% patients had digestive tract symptoms (PHQ15-D > 0, *N* = 329) and 57% are without digestive tract symptoms (PHQ15-D = 0, *N* = 440). Subgroups are classified by sex, age, drinking, appliance type, and treatment duration. HEI, OHIP-14, and PHQ15-D mean scores were significantly higher in females and patients with alcohol drinking habits. When the orthodontic appliance type changed from no appliance, clear alignment to labial appliances, the HEI, OHIP-14, and PHQ15-D mean scores increased as the discomfort increased. Notably, among groups with different genders, ages, appliance types, and treatment duration, the mean score of PHQ15-D exhibited a similar trend to HEI and OHIP-14, meaning that patients with more gastrointestinal symptoms have poor mental and oral health (Tables 1, 2A).

**TABLE 1:** Descriptive statistical analysis.

	PHQ15-D		HEI		OHIP-14	
(*N*)	Score	*P*-value	Score	*P*-value	Score	*P*-value
**Gender**						
Male (256)	0.73 ± 1.17	0.03*	3.90 ± 4.52	0.01*	8.54 ± 7.96	0.01*
Female (513)	0.87 ± 1.21		4.67 ± 4.62		9.86 ± 7.81	
**Age**						
≤15 years (140)	0.30 ± 0.63*^abc^*	*p^a^* = 0.00***	2.93 ± 3.54*^ab^*	*p^a^* = 0.00***	6.04 ± 6.66*^ab^*	*p^a^* = 0.00***
16∼18 years (100)	0.79 ± 1.19*^a^*	*p^b^* = 0.00***	4.84 ± 5.29	*p^b^* = 0.00***	7.97 ± 8.31*^cd^*	*p^b^* = 0.00***
19∼24 (229)	0.93 ± 1.20*^b^*	*p^c^* = 0.00***	4.67 ± 4.68*^a^*	*p^c^* = 0.00***	10.21 ± 7.23*^ac^*	*p^c^* = 0.00***
25∼34 (243)	1.02 ± 1.28*^c^*		4.76 ± 4.57*^b^*		11.20 ± 8*^bd^*	
≥35 years (57)	0.91 ± 1.47		4.79 ± 4.8		9.51 ± 9.02	
Mean age (±SD)	23.09 ± 9.549 years					
**Alcohol drinking**						
Yes (153)	1.15 ± 1.41	0.00***	5.79 ± 5.31	0.00***	11.99 ± 8.06	0.00***
No (616)	0.74 ± 1.12	4.07 ± 4.34			8.78 ± 7.71	
**Treatment duration**						
0 min (151)	0.59 ± 1.04*^a^*		3.91 ± 4.58		6.5 ± 6.92*^abcd^*	
≤1 min (31)	1.00 ± 1.39		4.94 ± 5.78		8.77 ± 8.2	
2∼6 min (51)	0.78 ± 1.06	0.01*	4.08 ± 3.55	0.34	8.86 ± 6.01*^a^*	0.00***
6∼12 min (149)	0.75 ± 1.18		4.47 ± 4.69		9.65 ± 7.57*^b^*	
12∼24 min (225)	0.8 ± 1.15		4.16 ± 4.2		9.69 ± 7.86	
≥25 min (162)	1.11 ± 1.34*^a^*		5.17 ± 5.04		11.86 ± 8.65*^d^*	
**Appliance type**						
Labial	0.90 ± 1.25*^a^*		4.58 ± 4.58		10.60 ± 8.1*^a^*	
Clear aligner	0.84 ± 1.15	0.04*	4.41 ± 4.66	0.22	8.96 ± 7.41*^b^*	0.00**
Without (Didn’t start)	0.59 ± 1.04*^a^*		3.91 ± 4.58		6.50 ± 6.92*^ab^*	

P-value < 0.05 was considered statistically significant. *P < 0.05, **P < 0.01, and ***P < 0.001. The values which has same superscript lower case letter have statistic difference.

**TABLE 2:** Scale analysis.

A	PHQ15-D		OHIP-14	
Scale HEI Score (*N*, percent)	Score	*P*-value	Score	*P*-value
Normal: ≤8 (631, 82%)	0.66 ± 1.06^abc^		8.26 ± 7.19^abc^	
Mild: 9∼12 (101, 13.2%)	1.48 ± 1.34^a^	*P^a^* = 0.01*	14.23 ± 8.68^a^	*P^a^* = 0.01*
Moderate: 13∼16 (17, 2.2%)	1.65 ± 1.23^b^	*P*^b^ = 0.01*	14.71 ± 6.96^bc^	*P*^b^ = 0.03*
Severe: ≥17 (20, 2.6%)	1.9 ± 1.97^c^	*P*^c^ = 0.03*	17.25 ± 9.33^c^	*P*^c^ = 0.03*

**B**	**HEI**		**OHIP-14**	
		
**Scale PHQ15-D** **Score (N)**	**Total**	**Depression**	**Anxiety**	**Score**

With symptom (329)	6.10 ± 4.89	3.95 ± 2.99	2.82 ± 2.37	12.48 ± 8.13
Without symptom (440)	3.15 ± 3.90	1.74 ± 2.25	1.41 ± 1.90	7.13 ± 6.74
*p*-value	0.00**	0.00**	0.00**	0.00**

P-value < 0.05 was considered statistically significant. *P < 0.05, **P < 0.01, and ***P < 0.001. The values which has same superscript lower case letter have statistic difference.

### Scale analysis

According to the PHQ15-D score, patients with and without digestive tract symptoms were divided into two groups to compare the mean scores of HEI, anxiety, depression, and OHIP-14. The anxiety and depression scores were the sums of the corresponding item scores on the HEI scale, respectively. The result revealed that the group with gastrointestinal symptoms exhibited much higher HEI and OHIP-14 mean scores than the group without such symptoms, as well as the score for anxiety and depression (Table 2B).

### Correlation analysis

The Spearman correlation analyses revealed that the OHRQoL and mental health (anxiety and depression) positively correlate with digestive tract conditions in orthodontic patients. The Spearman coefficients between PHQ-15-D and HEI and with OHIP-14 were 0.36 (*P* = 0.00^***^) and 0.36 (*P* = 0.00^***^), respectively, and the coefficients between PHQ-15-D and anxiety and with depression were 0.351 (*P* = 0.00^***^) and 0.34 (*P* = 0.00^***^), respectively. The regression analysis revealed significant correlations, demonstrating that an increase in PHQ15-D by a score increased the mean score of HEI and OHIP-14 by an average of 2.13 and 3.97, respectively.

### Chained medication model

**FIGURE 1:**
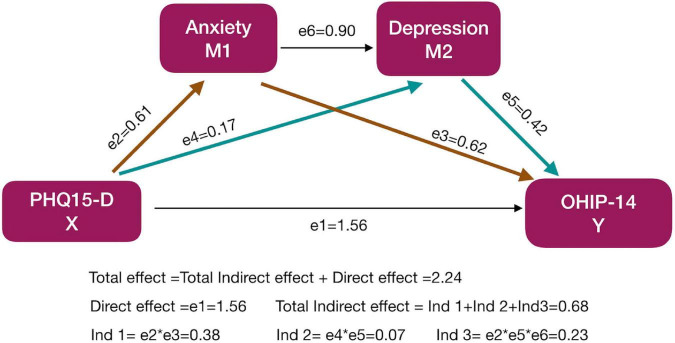
The schematic diagram of the chain intermediary analysis model. The influence of anxiety and depression on the relationship between digestive conditions and alcohol was evaluated. Regression analysis was performed for every two variables to obtain different effect coefficients (e1, e2, e3, …), and the indirect effect coefficient values were further calculated for the different pathways. Different colours were used to better display the equations. The yellow arrows (e2, e3) were effect coefficients included in Ind 1, the green arrows (e4, e5) in Ind 2.

The results of the chain mediation model revealed that none of the 95% confidence intervals included 0, and the model exhibited partial mediation effects. The total effect is 2.24, the direct effect is 1.56, and the indirect effect is 0.68, accounting for 30% of the total effect. Furthermore, the mediating effect of anxiety is 0.38, accounting for 56% of the total indirect effect. The mediating effect of depression is 0.07, accounting for 10% of the total indirect effect. The mediation effect of anxiety-depression is 0.23, accounting for 34% of the total indirect effect. The above results suggest the following: (1) anxiety and depression mediate the relationship between gastrointestinal conditions and OHRQoL and (2) changes in the digestive tract status of orthodontic patients affect OHRQoL mainly through the mediation variable of anxiety (Table 3 and Figure 1).

**TABLE 3:** Chained mediation model.

	Effect	SE	*p*	95% CI (LLCI, ULCI)
Total effect	2.24	0.22	0.00***	(1.80, 2.68)
Direct effect	1.56	0.23	0.00***	(1.10, 2.00)
Indirect effect				
Total	0.68	0.11	0.00***	(0.47, 0.92)
Ind 1	0.38	0.14	0.00***	(0.12, 0.66)
Ind 2	0.07	0.04	0.00***	(0.01, 0.15)
Ind 3	0.23	0.10	0.00***	(0.04, 0.45)

Ind 1, PHQ15-D-Anxiety-OHIP-14; Ind 2, PHQ15-D-Depression-OHIP-14; Ind 3, chained mediation path, PHQ15-D-Anxiety-Depression-OHIP-14; Total effect = Total indirect effect + Direct effect; Total indirect effect = Ind 1 + Ind 2 + Ind 3. P-value < 0.05 was considered statistically significant. *P < 0.05, **P < 0.01, and ***P < 0.001.

## Discussion

Several studies have reported that patients with malocclusion undergoing orthodontic treatment have more digestive tract symptoms and psychological problems, such as anxiety and depression, which reduce OHRQoL. In our study, we used a large sample size and systematically investigated the sex, age, and treatment effects on psychological status, digestive tract symptoms, and OHRQoL. Moreover, we evaluated the mediation relationships among psychological status, digestive tract symptoms, and OHRQoL and discovered that anxiety and depression mediate the relationship between digestive tract conditions and OHRQoL.

In line with a previous study (Piccinelli and Wilkinson, 2000), our results demonstrated that females and patients with drinking habits had higher anxiety and depression scores and more severe digestive tract symptoms. During orthodontic treatment, the diet habits and oral function change a lot in patients. A study by Aleksandrova et al. (2017) revealed that diet plays a vital role in regulating intestinal microbiota and influencing epigenetic changes. Changes in dietary habits can lead to different compositions and distribution of intestinal flora, which are often associated with digestive tract diseases, such as inflammatory bowel diseases and irritable bowel syndrome (Zannini and Arendt, 2018). A study has illustrated that more gastroesophageal reflux symptoms in orthodontic patients are observed because of decreased masticatory function (Johal et al., 2015). Long-term wear of orthodontic brackets increases the incidence of ulcers and may cause discomfort symptoms such as pain after orthodontic force, which will also affect the chewing function and eating habits of orthodontic patients. Furthermore, inhibition of masticatory function would reduce salivation exposure, impaired bolus formation, inadequate gastric acid production, and digestive disorders (Zhang et al., 2018). Our results revealed that patients with labial appliances and longer treatment durations with more discomfort have higher PHQ15-D scores, consistent with previous findings.

Physical health is strongly associated with mental health. Physical symptoms, such as digestive symptoms and pain, can cause psychological problems and externalize mental health states, such as anxiety and depression. Our study demonstrated that patients with digestive tract symptoms had worse psychological states than normal patients. Moreover, correlation analysis revealed that gastrointestinal conditions were positively correlated with mental health status. Psychological status was correlated with oral health, and they affected each other. Recently, the influence of gut microbiota on mental health and the nervous system has become one of the most attractive topics in microbiology (Bear et al., 2020). Valles-Colomer et al. (2019) reported a positive correlation between the quality of life and the ability of gut microbes to assemble into the neurotransmitter dopamine, suggesting that the microbiome can influence mental health. All the evidence combined with our findings highlight the vital interactions between mental health and gut microbiota, even in patients with malocclusion.

Our findings identified that digestive tract conditions are positively related to psychological and oral health status. According to gut-brain axis studies and psychosomatics, changes in digestive tract conditions can directly affect mental and oral health status, but they can also have an indirect psychological impact on oral health (Clarke et al., 2008; Foster and McVey, 2013). We hypothesized that psychological status (anxiety and depression) might mediate the relationship between gastrointestinal conditions and OHRQoL in orthodontic patients. Anxiety and depression often exist together clinically and have similar clinical presentations and somatization symptoms. The chain mediator analysis model can explore the mediation role of anxiety and depression in the relationship between gastrointestinal status and OHRQoL. The results showed that anxiety and depression mediated the relationship between digestive tract status and quality of life and that anxiety accounted for 56% of the total mediating effect. Thus, changes in masticatory function and eating habits in orthodontic patients may result in dysregulation of intestinal flora and gastrointestinal symptoms, and intestinal flora would further affect patients’ emotions by secreting related cytokines to increase the sensitivity of orthodontic patients to oral symptoms and reduce the OHRQoL. Our study about interventions for digestive tract problems of orthodontic patients may assist a consequential reduction in the incidence of psychological problems and improve the satisfaction of orthodontic therapy outcomes in the future, which is neglected generally in clinical therapy.

Some limitations are present in our study. First, a cross-sectional study cannot identify causal effects, and longitudinal studies must confirm these findings. Second, although we applied the chained mediation model to reveal the mediation effects of psychological status on digestive tract conditions and OHRQoL, there is still a lack of direct evidence like that found in animal models about the relationship among gut bacteria, psychological health, and neurological system, and further research to explore the mechanisms is required at the level of cells, tissues, and genes.

## Conclusion

Using a large sample size of patients with malocclusion, our study revealed the following conclusions: (1) In orthodontic patients, the digestive tract conditions are positively related to psychological and oral health status. (2) Anxiety and depression mediated the relationship between gastrointestinal conditions and OHRQoL, particularly for anxiety, which plays a predominant mediation role. (3) In addition to psychological status, supervision for digestive tract symptoms in orthodontic patients is required in future clinical practices. Our findings may improve the future clinical care and treatment of orthodontic patients.

## Data availability statement

The original contributions presented in this study are included in the article/supplementary material, further inquiries can be directed to the corresponding authors.

## Ethics statement

The studies involving human participants were reviewed and approved by the Human Research Ethics Committee of Sichuan University (WCHIRB-D-2020-209). Written informed consent to participate in this study was provided by the participants and their legal guardian/next of kin.

## Author contributions

WD: conceptualization and guidance. L-ZD and W-ZH: methodology. XT and Y-HL: analysis and data curation. DP: investigation. XT: writing – original draft preparation and review and editing. X-LH and S-FD: supervision. All authors contributed to the article and approved the submitted version.

## Abbreviations

OHRQoL: oral health-related quality of life
OHIP-14: oral health impact profile-14
PHQ15-D: digestive tract condition investigated by Patient Health Questionnaire-15
HEI: Hua-Xi emotional-distress index.
